# Personhood and Moral Status of The Embryo: It’s Effect on Validity of Surrogacy Contract Revocation according to Shia Jurisprudence Perspective

**DOI:** 10.22074/ijfs.2017.4970

**Published:** 2017-09-03

**Authors:** Saeid Nazari Tavakkoli

**Affiliations:** Department of Jurisprudence and Principles of Islamic Law, Faculty of Theology and Islamic Studies, Tehran University, Tehran, Iran

**Keywords:** Personhood, Moral Status, Embryo

## Abstract

**Background:**

One of the most controversial issues related to the human embryo is the determination of the moment when an embryo is considered a human being and acquires a moral status. Although personhood and moral status are frequently mentioned in medical ethics, they are considered interdisciplinary as concepts that shape the debate in medical law (fiqh) since their consequences are influential in the way which the parents and other individuals behave towards the embryo.

**Materials and Methods:**

This analytical-descriptive research gathered relevant data in a literature search. After a description of the fundamentals and definitions, we subsequently analyzed juridical texts and selected one of the viewpoints that regarded the surrogacy contract revocation.

**Results:**

The surrogacy contract is a contract based upon which two sides (infertile couple and surrogate mother) involved in making the contract are obligated to fulfill its terms. Therefore, contract revocation can be surveyed from three perspectives: mutual revocation (iqala), legal unilateral wills (khiar al-majlis, khiar al-ayb), and contractual wills (khiar al-shart).

**Conclusion:**

Revocation of a surrogacy contract either by the genetic parents, surrogate or the fertility clinic is allowed by Muslim jurists only when the embryo lacks personhood. Based on Islamic teachings, the termination of a surrogacy contract in and after the sixteenth week of pregnancy, when the embryo acquires a human soul (ensoulment), is not allowed. However religious thought emphasizes the moral status of the fetus before the sixteenth week and states that optional termination of the surrogacy contract is not permitted while the fetus becomes a human being.

## Introduction

The desire to have children is associated with the creation of human beings. Surrogacy is one method used by infertile couples who wish to have a baby. The first legal surrogacy agreement was enacted in the mid-1970s. The first paid traditional surrogacy arrangement was conducted in the United States in 1980. In 1983, the first successful pregnancy occurred via egg donation. This event later led to the first gestational surrogacy in the United States in 1985 (1, 2). In a study reported the first surrogate gestational pregnancy that resulted in heated debates in the UK. In 1985, the UK established the Surrogacy Law (3) which permitted surrogacy under special circumstances. Therefore, the UK became one of the few European countries where a surrogacy contract was permitted under specific circumstances. In 1959, Patrick Steptoe and Professor Robert Edwards initially introduced the surrogacy contract in Europe. Finally, in 1990, the UK passed the Human Fertilization and Embryology Act (the 1990 Act), which was revised in 2008 (4). However, since the United States has legally allowed commercial surrogacy contracts, the majority of these contracts reportedly occur in this country.

In Iran, the first infertility center was inaugurated in Yazd in 1989. The Gamete and Embryo Donation law (5) was passed in 2003 which has permitted gestational surrogacy for infertile couples. Gestational surrogacy is when *in vitro* fertilization (IVF) is performed after aspiration of the husband's sperms and wife's eggs. The zygote or embryo is subsequently transferred into the womb of the surrogate mother to naturally spend his early development. After birth, the surrogate then gives the child to the intended (genetic) parents. Use of a surrogate and surrogacy during pregnancy is divided into several classes based on the presence or absence of a genetic link between the surrogate and the intended parents. The most common type of surrogacy is “full surrogacy" that has no genetic link (6). Full surrogacy is used when there is a fertility disorder or dysfunction such as habitual abortion or another unknown problem. In this case, the ovum is fertilized by husband’s sperm in vitro and the embryo is subsequently transferred into the surrogate uterus (7, 8).

## Materials and Methods

This analytical-descriptive research gathered the relevant data as a literature search. After describing the jurisprudents’ fundamentals, we subsequently analyzed the Shiāat fiqh sources. This was not a clinical trial, hence no need existed for patient consent or Ethical Committee approval.

### Validity of the surrogacy contract

The surrogacy contract is an arrangement between an infertile couple and a woman who agrees to conceive with reproductive methods using their embryo. Based on this contract, the surrogate agrees to accept transfer of the embryo from the genetic parents to her uterus. The surrogate accepts responsibility to maintain the pregnancy and perform conventional measures for fetal growth until the child is born. She agrees to refrain from risky, harmful behaviors that affect fetal growth and consents to return the child to the infertile (genetic) couple after its birth. There are two, legal surrogacy contracts: commercial surrogacy and altruistic surrogacy. If the genetic parents (with the surrogate’s agreement) guarantee compensation for her involvement, this contract is termed a commercial surrogacy. However, altruistic surrogacy is when the surrogate accepts to become pregnant and deliver the baby to the parents without any financial compensation and only for charity motives. The surrogacy contract has three essential participants: surrogate mother, infertile couple (sperm owner and ovule owner) and the fertility clinic. The fertility clinic is the therapist center in which necessary actions for fertilization of the infertile couple’s sperm and ovule is performed which results in its subsequent placement into the surrogate mother’s uterus by a necessary medical procedure.

This agreement is legal according to article 10 of the civil law, “freedom of contracts”, and permitted (ebahah principle) (9) since it is not contrary to the law. This contract is valid and effective, and Commercial and Altruistic of this contract has not effect on its being lawful (javaz) or validity (sehhat). Although the principle of autonomy sovereignty and freedom of contracts can prove the legality of a surrogacy contract, of note, such an argument may not suffice since the sanctity of using reproductive organs outside the context of marriage can be the primary obstacle to the effect of the autonomy sovereignty. The need to respect the precautionary principle (asle ehtiat) in such cases is an obstacle to perform freedom of contracts principle (ebahah principle). Thus, the validity of a surrogacy contract faces a fundamental problem (10).

### Contract revocation due to cancellation by the parties of the contract

Based on the principles of Islamic Law and jurisprudence, the contract can be revoked (11). It is possible only due to the application of one of the legal wills (khiar; khiar al-majlis, khiar al-ayb) or contractual wills (khiar al-shart). A surrogacy contract is a contract based upon which the two sides involved in making the contract are obligated to fulfill its terms unless a health risk or problem occurs for the surrogate mother. Therefore, contract revocation can be surveyed from two perspectives:

#### Mutual revocation

Iqala is one of the ways to terminate the commitments whereby the contract can be annulled by mutual agreement of the parties (12) and avoid its sequence effects in the future. Annulment of a surrogate contract by iqala is examined in three assumptions.

#### Mutual revocation before starting the process

If the genetic parents, surrogate mother, and fertility clinic agree upon creation of the embryo and its transfer to the surrogate’s uterus and they sign a contract with the mediation of the fertility clinic but no embryo has yet formed *in vitro*, can the contract be revoked by cancellation of each of the parties involved? There is no doubt that the surrogacy contract is a continuous contract whereby, before the process (the issue of contract), mutual revocation (iqala) exists (13). However, regarding the fact that mutual revocation (iqala) is permissible according to mutual agreement of the parties (tarazi), can either of the parties involved (the genetic parents, surrogate mother or fertility clinic) alone dissolve the contract or is it dependent on mutual agreement of all the members?

If, after signing the contract, the genetic parents are not inclined to give their sperm and egg to the fertility clinic to form the embryo during the process of fertilization, then the fertility clinic and the surrogate mother have no commitment to the terms of the contract. Furthermore, if the fertility clinic is not willing to perform IVF and its transfer to the surrogate’s uterus, the surrogate’s commitment to the contract is meaningless. Finally, if the surrogate refrains from pursuing the contract and she does not allow the fertility clinic to implant an infertile couple’s IVF embryo into her uterus, no issue will remain for the surrogacy contract. Ethically she has a duty for the contract’s execution, but she cannot be forced to uphold the contract. There is a longitudinal relationship between the effective parties’ demands and their commitment to this contract. Each party’s commitment is an essential condition for contract execution and no action can be considered a sufficient condition, however the commitment of all parties toward the terms of the contract is a sufficient condition for its performance. In this case when one of the parties is not inclined to continue the surrogacy contract and decides to terminate the contract, it will automatically be terminated due to the loss of its subject. It is obvious that this revocation results from failure to commit from each of the parties, and is not due to iqala. Therefore, each party’s will in the surrogacy contract is alone sufficient for contract revocation, either with iqala that includes the mutual consent of all parties or not to commit to contract terms that would lead to termination of the contract.

#### Mutual revocation after egg cell formation and before implantation in the surrogate’s uterus

If the fertility clinic performs IVF after the request and consent of the genetic parents and agreement of the surrogate, the contract revocation can be surveyed from three perspectives: i. The decision made by one or both genetic parents about not having a child, ii. Refusal of the fertility clinic to continue the process, or iii. Refusal of the surrogate to lend her uterus for the embryo’s implantation. The common point in these three assumptions is that cancellation of each of the parties involved will destroy the human embryo. If the created embryo is not used for scientific research or donated to other infertile couples or frozen for the genetic parents’ future use, will this be embryo destruction or prevention of its life. Is this permissible or not? In the first and second cases, the request of contract revocation from the genetic parents or fertility clinic, and lack of commitment to contract’s terms results in destruction of the embryo. However, the third case that the surrogate refuses to lend her uterus does not prevent the embryo from surviving because the embryo can survive *in vitro* before being implanted in another uterus. Although, finding other appropriate surrogates takes time and the golden time for implantation could be lost.

Permitting or not permitting the destruction of a human embryo is based upon two issues: the personhood of the embryo and the condition of embryo placement in the uterus. If we consider the zygote as a human being after its formation, then we should accept that it possesses human dignity and therefore each action which leads to its destruction will be unlawful. Moreover, if we accept the validity of embryo placement in the uterus, not in terms of its subject matter, but only in terms of its method (14), the refusal of genetic parents, the fertility clinic, or both to follow surrogacy contract terms is still not allowed since it will destroy a human being. However if before ensoulment, the fetus or human embryo is not considered to be a human being or we consider its placement in the uterus as a subject, it will be allowable. Because the prevention to perform the contract’s terms and it’s termination does not cause the destruction of a human being and this beings doesn’t place in surrogate’s uterus. The Qur'an considers two stages of embryo development: before and after ensoulment (15).

Thus, human life begins with its ensoulment, as its revocation occurs with separation of the soul from the body (16, 17). There is no doubt that a human embryo after ensoulment is considered a human being; any action that leads to its death (abortion) is the killing of a living creature (manslaughter) and this is an unlawful action. Whoever performs this action, regardless of whether it is the mother, father or any other person must pay blood money for killing a full-grown man in addition to eternal punishment. Nevertheless, this judgment does not imply that the human embryo lacks personhood before ensoulment (i.e., before the sixteenth week, from the 4th month onwards) and therefore it will ethically and religiously be allowed to perish since the Qur'an considers beginning of human creation from the moment of egg cell (Notfa) creation (18).

The egg cell does not refer only to male sperm but it refers to the fertilized male sperm and female egg (zygote) (Notfa Amshaj) (19). Since the egg cell will fail to humanize before it is implanted in the uterine wall, God considers implantation in the uterine wall as the precondition for human creation (according to the author’s understanding of this verse) (20). Accordingly, after implantation, after the second week onwards, the human being will undoubtedly be formed. The question is whether any human person exists before this period? The simplest interpretation is to say that an egg cell is not considered a human before reaching this stage (i.e., from the baseline week until the second week). In other words, when the egg cell is only an egg cell (21).

However, a human can be defined since the formation of the egg cell, that is, shortly before reaching the implantation stage. A proof of this claim, in addition to narratives of the infallible Imams (22), is the understanding of Muslim jurists (foqaha). Therefore, the request for surrogacy contract dissolution is an unlawful (haram) act since it causes the death of a potential life. In the third case where the genetic parents and the fertility clinic are committed, the parents provide sperm and ovule to the institution and the institution creates a germ cell and embryo, but the surrogate has requested to terminate the contract. She does not lend her uterus for implantation despite the previous agreement. What should be done?

According to the principle of autonomy sovereignty and a human’s inherent genetic ownership of his/her body’s integrity (23, 24) as well as the lack of legal hindrance to terminate the contract in terms of self-preservation, the contract is terminated by the request of the surrogate and consent of the other parties of the contract. Because the duty of self-preservation, is not a duty that only the surrogate should notice; and the other women could not have a mediating role and they could not perform it until when the surrogate cancel it, potential human is destroyed. But if there is no consent by the other parties of the contract, there will not be iqala and the surrogate must fulfill her commitment. Until the surrogate does not lend her uterus, it cannot act on the contract terms and create a human. On the other hand, there is not a binding force of the surrogate to the nine months of pregnancy acceptation and the surrogacy contract is automatically terminated. It is clear that the surrogate mother is responsible to compensate for the losses imposed on the genetic parents or fertility clinic since contract revocation by the person who has requested it does not negate compensation for the losses that result from revocation. As in the previous two cases, contract revocation by the genetic parents or the fertility center does not negate the compensation for the losses imposed on the surrogate or the other party.

#### Mutual revocation after implantation in the surrogate’s uterus

If the genetic parents, the fertility clinic, and the surrogate fulfill their obligations based on the surrogacy contract and the *in vitro* formed embryo is injected into the surrogate's uterus can the parties involved refuse to fulfill contractual provisions (terms) afterwards? Contract revocation by the genetic parents or the surrogate means that they are not inclined to continue the process of pregnancy. As a result, the process of pregnancy should be terminated. The existence of the right to revocation for each in this assumption is subject to determining the time of the embryo’s personhood, since the embryo has been settled in the surrogate's uterus. If we consider the human embryo as a person even before the sixteenth week, neither the genetic parents nor the surrogate has the right to annul the contract because taking into consideration their rights for revocation conflicts with the right of the embryo's life. However, if we do not consider the human embryo as a person until ensoulment, then we can possibly consider the right of revocation for both the genetic parents and surrogate.

Following these explanations, it is clear that no right of revocation exists for the parties involved in the contract after ensoulment because the embryo in the surrogate’s uterus, though not a property, belongs to all three parties involved: genetic parents, surrogate, and the embryo itself (although the fetus is not directly a party for the contract, but by performance of the contract terms and its creation by the fertility clinic, it will have the right to life). Therefor neither the mother nor father can use their hypothetical right to annul the contract. In addition, their agreement upon revoking the contract, assuming the agreement of the surrogate, will not be a reason for revocation since it is in contrast with the right of an embryo’s life. The right of life precedes any other right that negate that life, except in cases where not allowing the embryo to live is permitted, such as the priority of the mother’s life over the embryo’s life if the mother’s life is saved by an abortion (25, 26). Hence, one can claim that malformed or patient fetus abortion is not lawful after ensoulment or 16 weeks when the possibility exists for a viable birth or survival. Accordingly, use of mutual revocation (iqala) by any of the parties involved to annul the surrogacy contract is permitted only when the obligation to contractual provisions has not yet started or if it has started, the embryo has not been implanted in the surrogate’s uterus.

### Applying option

Option (khiar) in Islamic texts refers to the ability (governance) of one or two parties involved in contract revocation (27). The existence of the option of revocation (khiar al-faskh) for each of the effective parties involved in a surrogacy contract can be investigated from four perspectives:

#### Mention of the right to revocation in the contract

Mentioning the right to revocation in the contract and its application does not differ from what has been previously mentioned about mutual revocation (iqala) according to legal-jurisprudent (fiqh) texts.

#### Option of violation from a conduct’s condition

The conduct's condition refers to the obligation of either one party of the contract, both parties or a person outside the contract to do something which can be either within the regulations of the contractual provisions or beyond its provisions (28). Within the contract each of the effective parties is obligated to do something. For example, the obligation of the genetic parents to financially support the surrogate and pay for medical expenses related to surrogate's pregnancy; the surrogate's obligation to use essential nutrients necessary for growth of the fetus and refrain from alcohol and smoking (quitting the conduct), and have regular visits to a health center for checkups; or the obligation of the fertility center to monitor the health status of the surrogate and her embryo, and timely detection of fetal genetic diseases. However, if one side does not observe the contractual provisions, can the other side annul the surrogacy contract due to a contract violation?

Undoubtedly, violation from a conduct mentioned in the contract will lead to the option of revocation (khiar al-faskh) for the other side; however, as far as the surrogacy contract is concerned, the issues of embryo’s life and his personhood distinguish this contract from the others. If a violation from the condition mentioned in the surrogacy contract occurs before starting the procedure the possibility of revocation exists for other sides as well; however, if it occurs after implantation of the embryo (egg cell) in the surrogate’s uterus and if we consider personhood for the embryo, then the surrogacy contract cannot be annulled due to option of violation from the condition. This type of revocation means revocation of pregnancy and killing a human embryo which is an unlawful conduct based on Islamic regulations. However, it can force the violator to follow the condition by setting up some regulations; if any loss occurs, the person who has suffered a loss can force the violator to compensate for their loss. In cases where the condition is beneficial for the embryo, the genetic father, due to his right of custody of the embryo can act on behalf of the embryo and its rights.

#### Option of violation from a characteristic condition

A characteristic condition (shart e sefat) is one of the correct conditions in contracts that refer to one of the qualitative or quantitative features of the contract (12). The option of violation from a characteristic condition in the surrogacy contract refers to the clinic’s obligation to create a child with a specific attribute not realized in the child (embryo). For example, the clinic’s obligation to create a specific sex (boy or girl) or specific physical attributes by using genetic changes (specific eye color, ear and nose shape, or hair type, or a defined genetic trait) or to create specific number of children (single or twins). The fertility clinic’s commitment for creating an embryo with special characteristics is unmoral and illegal. However, if we assume that the fertility clinic is committed to this action, in those characteristics that their understandings are possible after the embryo’s growth in the surrogate mother’s uterus. In case of non-fulfillment of that characteristic, the surrogacy contract cannot be terminated due to a violation of the characteristic condition (shart e sefat). The prerequisite required to terminate the contract by the genetic parents is abortion or abandoning the child by entrusting it to the surrogate, and not accepting responsibility for the embryo-none of which are acceptable.

#### Option of deficiency

The fertility clinic is obligated to prevent creation of an embryo with physical abnormalities (paralyzed or disabled) in a timely manner by using diagnostic techniques based on previously accepted medical science and to prevent embryo development or at least tell the genetic parents about the condition of the embryo when such abnormalities occur. Then, the genetic parents can make a decision about the future of their child. However, if the fertility clinic, intentionally or mistakenly, does not fulfill its responsibility despite having the knowledge or necessary equipment and this oversight results in an abnormal embryo and at any stage of the embryo development, the genetic parents are not informed about the embryo’s defect, can the genetic parents based on the option of deficiency (khiar al-ayb) annul their contract with the surrogate?

Contract revocation based on option of deficiency (khiar al-ayb) is possible in two cases. The abnormality exists at the time of signing the contract; however, the other side is not aware of this abnormality or that another abnormality has occurred due to the previously existing abnormality after signing the contract. Accordingly, a surrogacy contract can be annulled based on option of deficiency (khiar al-ayb) when the genetic abnormality existed prior to signing the surrogacy contract with the fertility clinic in cases where the clinic, whether intentionally or mistakenly, did not disclose the abnormality to the parents that resulted in other physical abnormalities in the embryo due to a genetic disorder.

Nevertheless, the right to revocation cannot be given to the genetic parents at least after embryo ensoulment. However the contract has the financial burden and can be subject to the rules governing the termination of transactions. On the other hand, it is associated with human life. Other authors have discussed causes of malformed and pros and cons of patient fetus abortion (29). If a deformity or congenital defect in the fetus in the surrogate’s uterus is one of the indications listed in the Therapeutic Abortion Act approved 30 of May 2005, then the genetic parents can terminate the contract with the carrier and abort the embryo. However, if the deformity or congenital defect is not listed in the Act or the parents are informed after the sixteenth week, by citing the terminate authority, the contract can no longer be terminated and the embryo aborted.

Under such conditions, the genetic parents, fertility clinic, and surrogate should fulfill their contractual obligations towards the embryo. The genetic parents should accept their own deformed or abnormal child after birth. This custodial right cannot be ignored, as it is a natural-genetic right whereby the parents should take care of the child once their mutual relationship occurs (dependent on both parents and the child) (30). However, since taking care of a deformed child is financially more difficult than taking care of a normal child; therefore, the fertility clinic should compensate for this financial loss for not fulfilling its obligation to the issues of the surrogacy contract. The compensation rule exists, whether or not the fertility clinic had the obligation to create a healthy child. Based on common understanding, the contract relates to the creation of a healthy child unless the fertility clinic denies this responsibility (i.e., creating a healthy, not deformed child) in the contract.

## Discussion

Use of surrogacy is a new phenomenon which, like any other method, has raised questions that are mostly beyond the medical scope. These questions are legal and jurisprudent. One important question that relates to surrogacy is whether each of the parties involved in the contract have the right to terminate the surrogacy contract. The current article attempts to find an answer to this question and in case of cancellation by any member, what decision should be made regarding continuation of the pregnancy and the resultant baby. Each of the effective parties involved in the surrogacy contract can annul the contract prior to embryo formation without the consent of the other sides. The permission for surrogacy contract revocation by each of the effective parties involved in the contract after egg cell formation and prior to its implantation in the surrogate’s uterus is subject to not considering personhood for the embryo. The validity of embryo placement in the uterus does not permit its abortion. The permission for surrogacy contract revocation by each of the effective parties involved in the contract after embryo implantation in the surrogate’s uterus prior to ensoulment is subject to not considering personhood for the embryo. Surrogacy contract revocation by each of the effective contractual parties after embryo implantation in the surrogate’s uterus and its ensoulment is not permitted due to its inconsistency with the embryo’s right to live. Surrogacy contract revocation after embryo (egg cell) implantation in the uterus due to violation of one of the parties from the terms of the contract is not permitted. The violator is obligated to fulfill the condition; otherwise, he/she should compensate for the loss(es) imposed on the other parties.

In case of the clinic’s obligation to create a child with specific physical attributes which is not fulfilled, the surrogacy contract cannot be annulled based on a violation from shart e sefat. In this case, contract revocation by the genetic parents will result in an abortion or embryo abandonment (the burden of which would fall on the surrogate). If the fertility clinic is aware of a genetic disorder of the embryo prior to signing the surrogacy contract and does not, intentionally or mistakenly, tell the genetic parents about this defect, then the surrogacy contract can be annulled only before ensoulment (sixteenth week) based on option of deficiency (khiar al-ayb). Nevertheless, contract revocation after ensoulment is not permitted and the genetic parents should take custody of their abnormal/deformed child; however, the fertility clinic should compensate for the loss that resulted from not fulfilling its (clinic) obligations.

## Conclusion

According to the result of this paper there is a direct relationship between legitimacy of surrogacy contract revocation and moral status of the embryo. Surrogacy contract revocation either by the infertile couple, surrogate, or the clinic is allowed by Muslim Jurists only when the embryo lacks personhood. Based on Islamic teachings, termination of a surrogacy contract in and after the sixteenth week of pregnancy, when the embryo acquires human soul (ensoulment), is not allowed. However, religious teachings emphasize that the moral status of the fetus before 16 weeks that leads to optional termination of the surrogacy contract becomes is not permissible when the fetus becomes a human being.
